# Supplementary material for: Antibodies Targeting Human or Mouse VSIG4 Repolarize Tumor-Associated Macrophages Providing the Potential of Potent and Specific Clinical Anti-Tumor Response Induced across Multiple Cancer Types
Source: Int J Mol Sci. 2024 Jun 3;25(11):6160. doi: 10.3390/ijms25116160 (PMC11172757; doi:10.3390/ijms25116160)
Supplement: Supplementary file 1 [file ijms-25-06160-s001.zip › ijms-3013101-supplementary.pptx]

## Slide 1
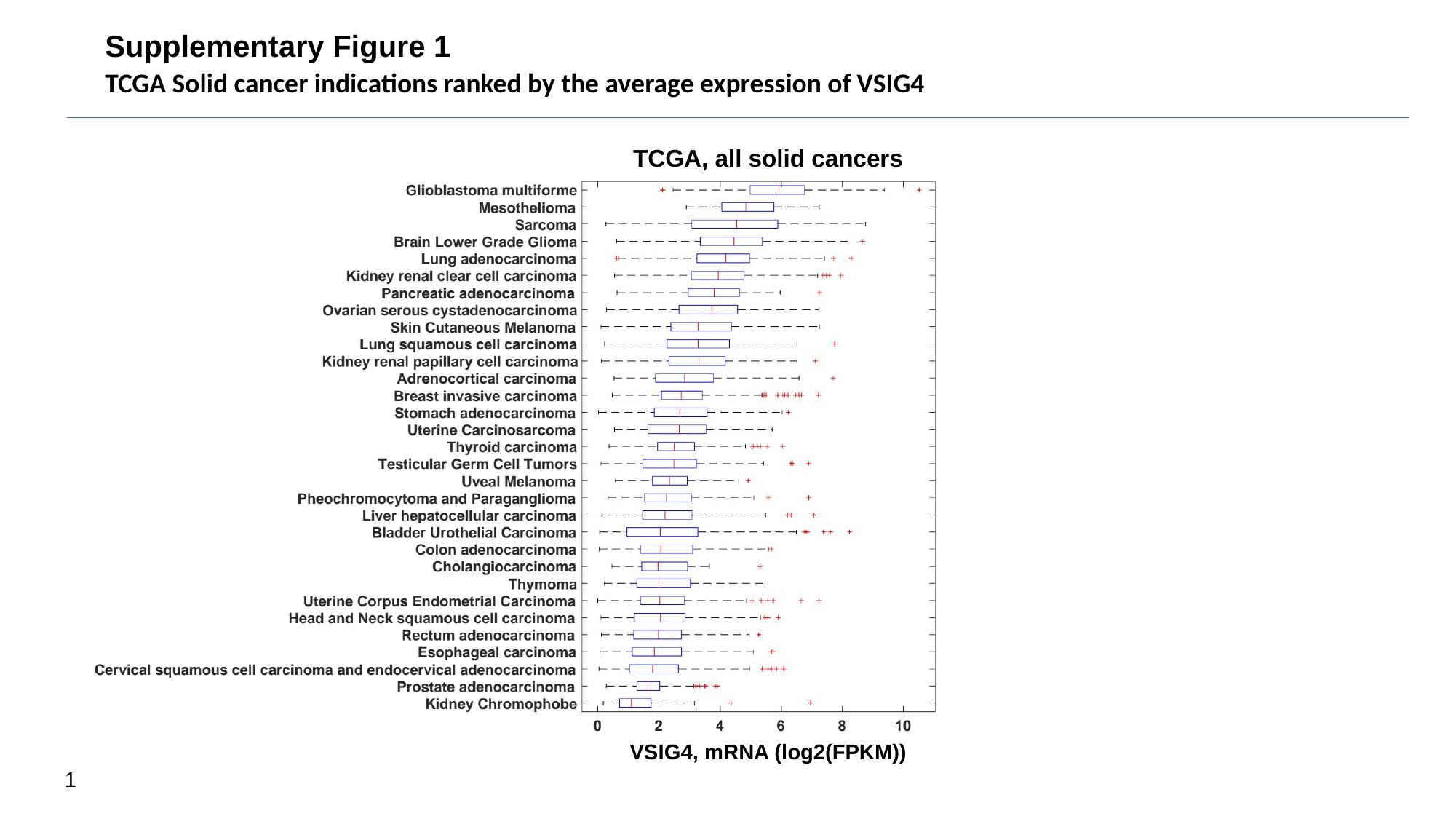

Supplementary Figure 1
TCGA Solid cancer indications ranked by the average expression of VSIG4
TCGA, all solid cancers
VSIG4, mRNA (log2(FPKM))
1

## Slide 2
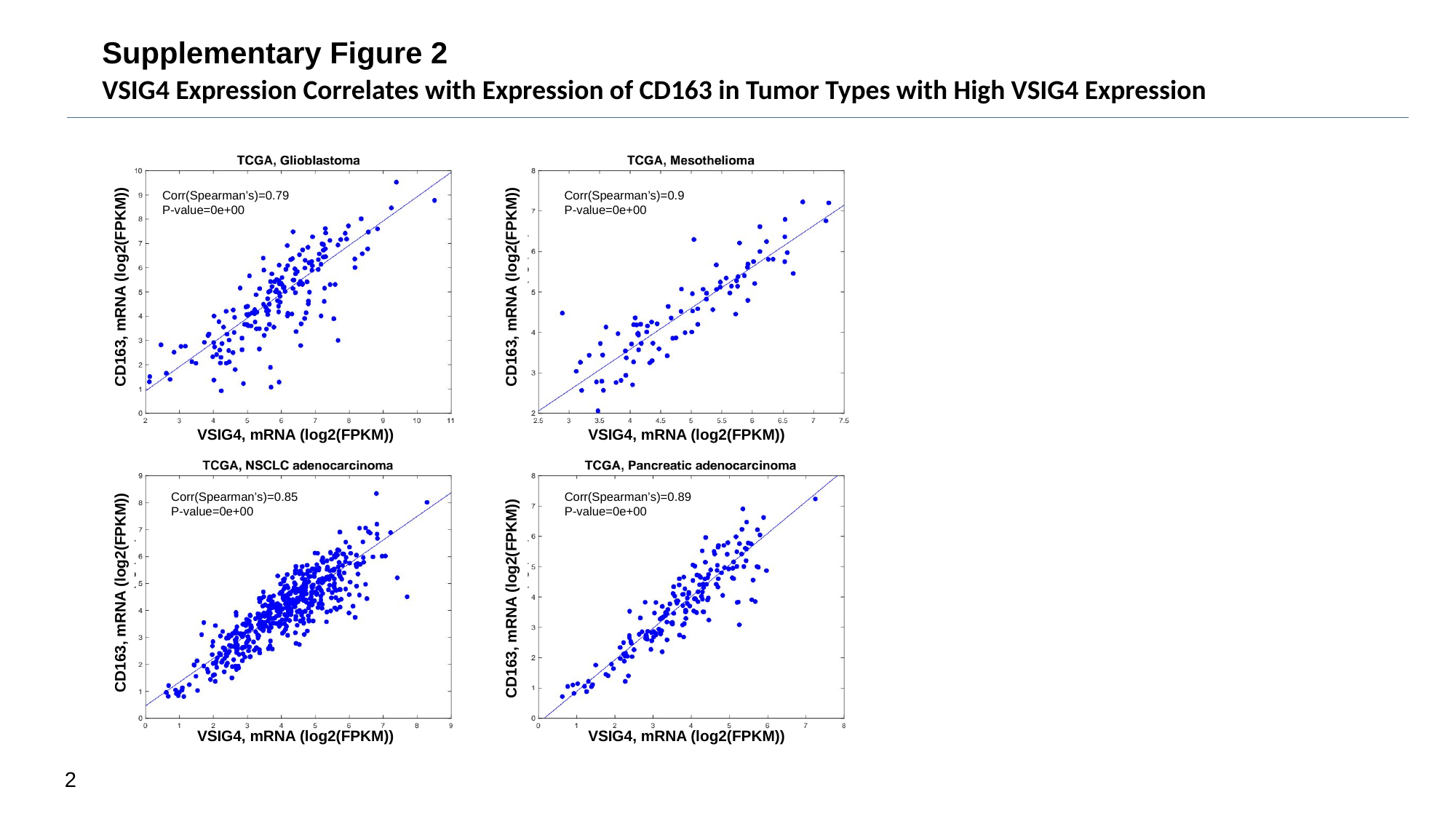

Supplementary Figure 2
VSIG4 Expression Correlates with Expression of CD163 in Tumor Types with High VSIG4 Expression
Corr(Spearman’s)=0.79
P-value=0e+00
Corr(Spearman’s)=0.9
P-value=0e+00
CD163, mRNA (log2(FPKM))
CD163, mRNA (log2(FPKM))
VSIG4, mRNA (log2(FPKM))
VSIG4, mRNA (log2(FPKM))
Corr(Spearman’s)=0.85
P-value=0e+00
Corr(Spearman’s)=0.89
P-value=0e+00
CD163, mRNA (log2(FPKM))
CD163, mRNA (log2(FPKM))
VSIG4, mRNA (log2(FPKM))
VSIG4, mRNA (log2(FPKM))
2

## Slide 3
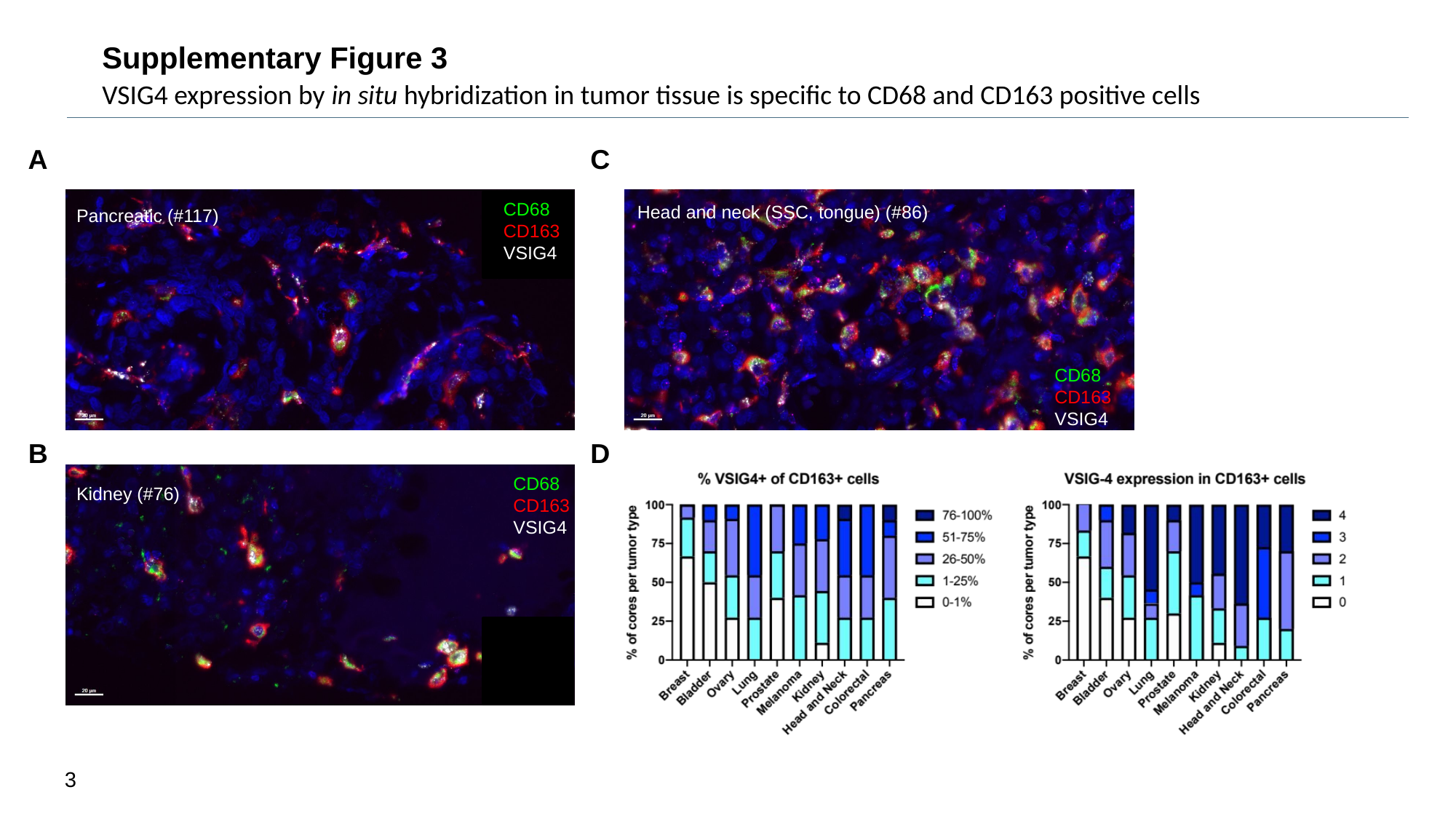

Supplementary Figure 3
VSIG4 expression by in situ hybridization in tumor tissue is specific to CD68 and CD163 positive cells
A
C
Pancreatic (#117)
Head and neck (SSC, tongue) (#86)
CD68
CD163
VSIG4
CD68
CD163
VSIG4
D
B
Kidney (#76)
CD68
CD163
VSIG4
3

## Slide 4
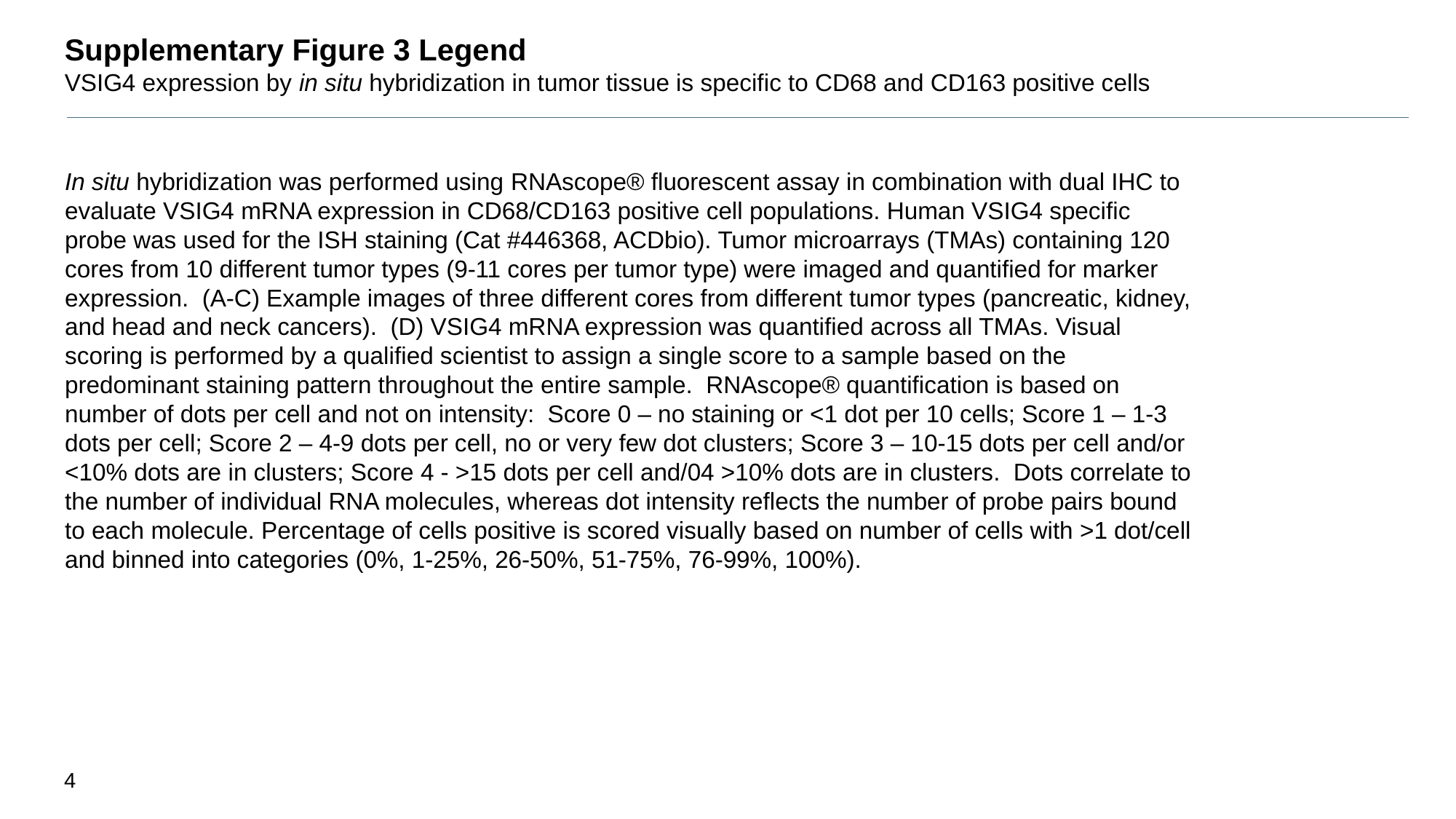

# Supplementary Figure 3 Legend VSIG4 expression by in situ hybridization in tumor tissue is specific to CD68 and CD163 positive cells
In situ hybridization was performed using RNAscope® fluorescent assay in combination with dual IHC to evaluate VSIG4 mRNA expression in CD68/CD163 positive cell populations. Human VSIG4 specific probe was used for the ISH staining (Cat #446368, ACDbio). Tumor microarrays (TMAs) containing 120 cores from 10 different tumor types (9-11 cores per tumor type) were imaged and quantified for marker expression. (A-C) Example images of three different cores from different tumor types (pancreatic, kidney, and head and neck cancers). (D) VSIG4 mRNA expression was quantified across all TMAs. Visual scoring is performed by a qualified scientist to assign a single score to a sample based on the predominant staining pattern throughout the entire sample. RNAscope® quantification is based on number of dots per cell and not on intensity: Score 0 – no staining or <1 dot per 10 cells; Score 1 – 1-3 dots per cell; Score 2 – 4-9 dots per cell, no or very few dot clusters; Score 3 – 10-15 dots per cell and/or <10% dots are in clusters; Score 4 - >15 dots per cell and/04 >10% dots are in clusters. Dots correlate to the number of individual RNA molecules, whereas dot intensity reflects the number of probe pairs bound to each molecule. Percentage of cells positive is scored visually based on number of cells with >1 dot/cell and binned into categories (0%, 1-25%, 26-50%, 51-75%, 76-99%, 100%).
4

## Slide 5
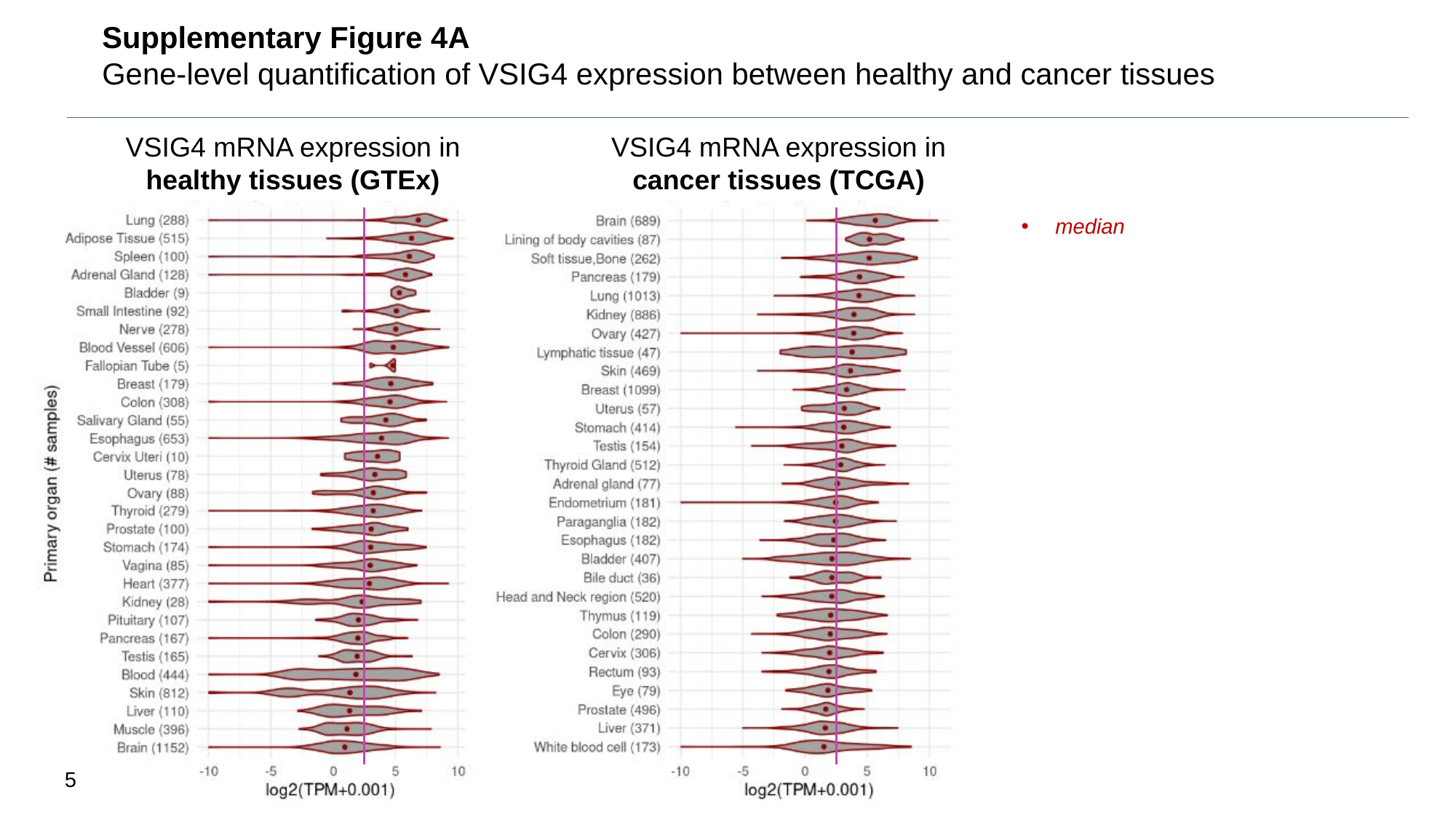

Supplementary Figure 4A
Gene-level quantification of VSIG4 expression between healthy and cancer tissues
VSIG4 mRNA expression in healthy tissues (GTEx)
VSIG4 mRNA expression in cancer tissues (TCGA)
median
5

## Slide 6
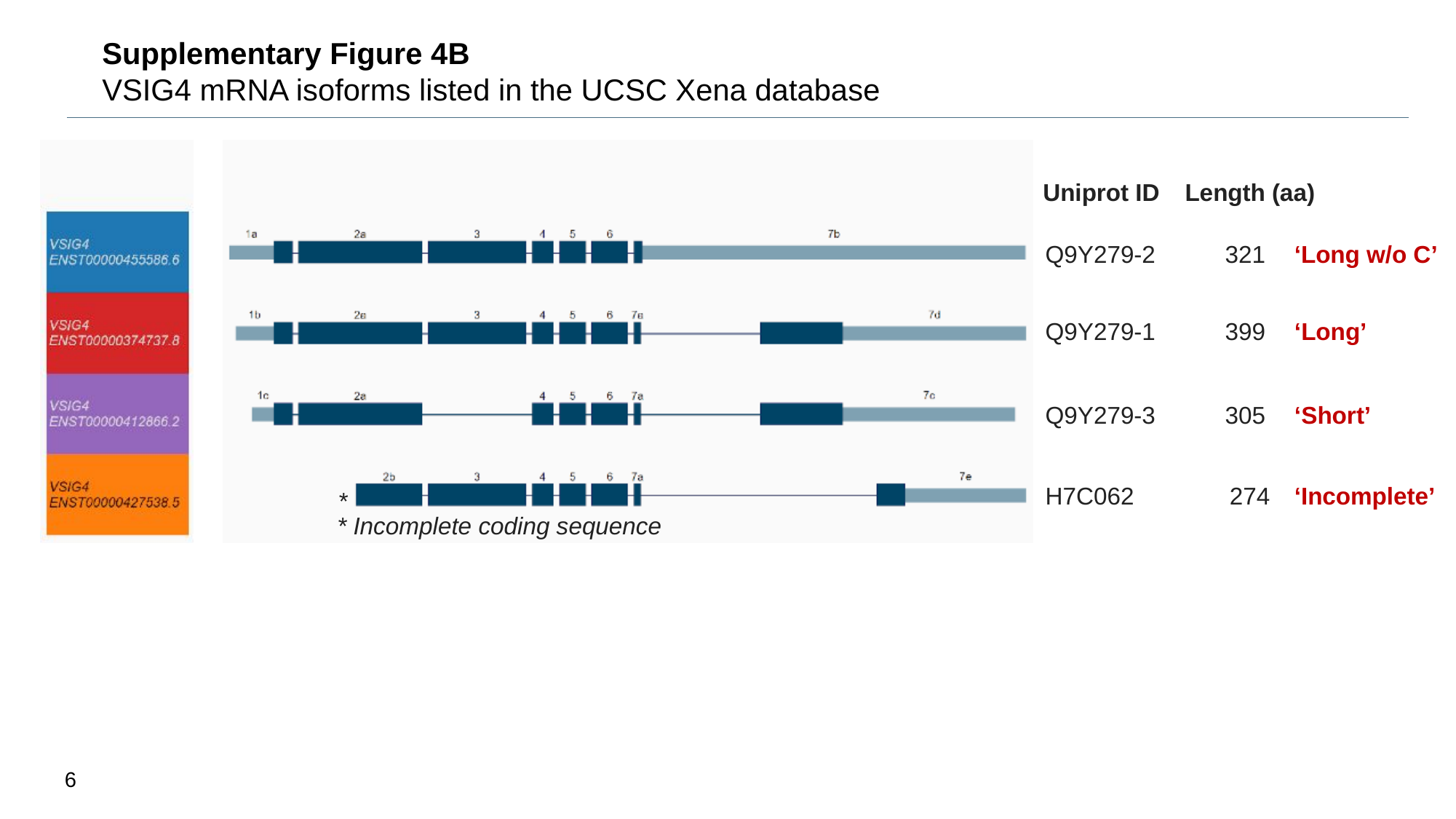

Supplementary Figure 4B
VSIG4 mRNA isoforms listed in the UCSC Xena database
Uniprot ID
Length (aa)
Q9Y279-2
321
‘Long w/o C’
Q9Y279-1
399
‘Long’
Q9Y279-3
305
‘Short’
H7C062
274
‘Incomplete’
*
* Incomplete coding sequence
6

## Slide 7
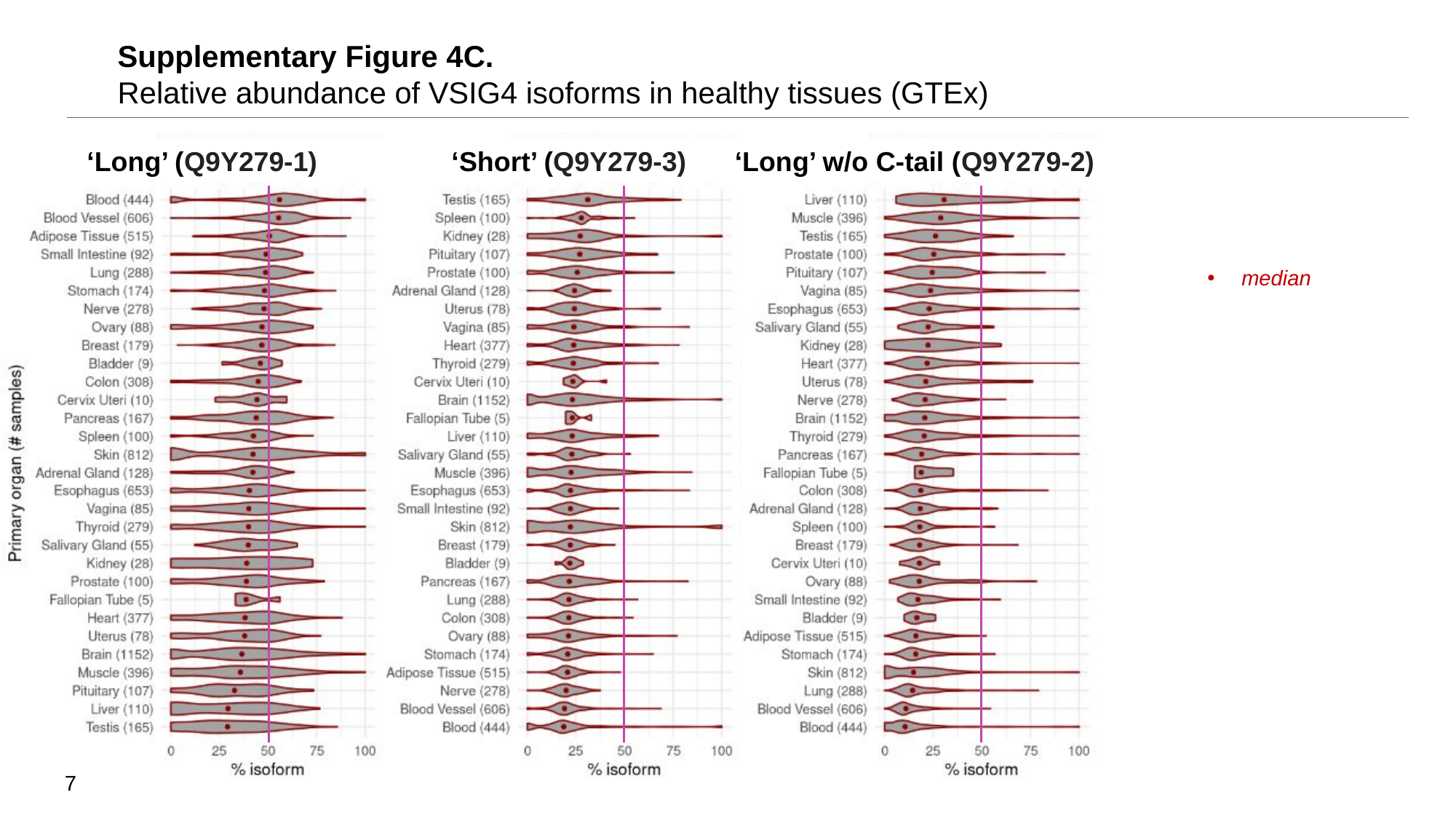

Supplementary Figure 4C.
Relative abundance of VSIG4 isoforms in healthy tissues (GTEx)
‘Long’ (Q9Y279-1)
‘Short’ (Q9Y279-3)
‘Long’ w/o C-tail (Q9Y279-2)
median
7

## Slide 8
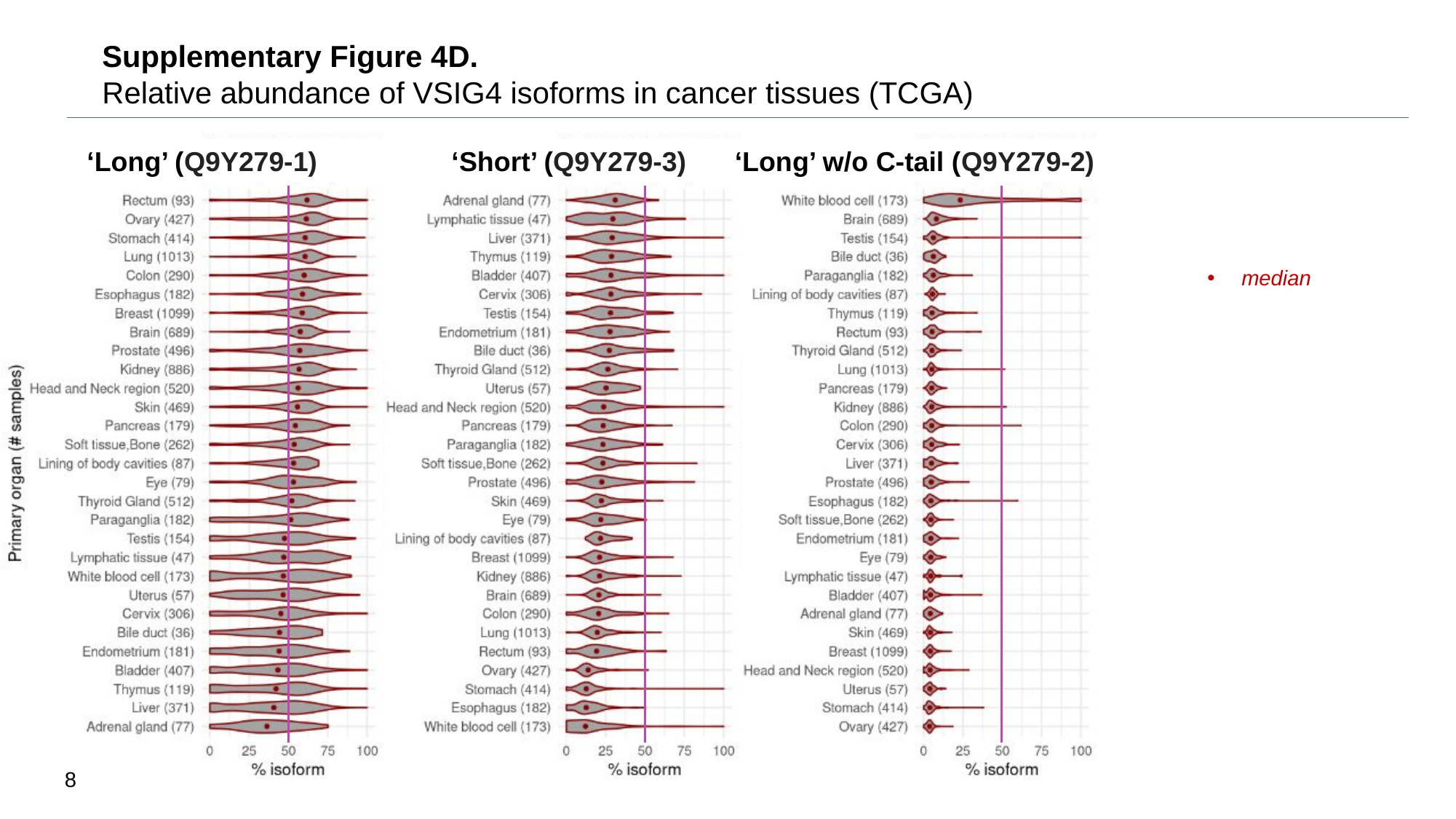

Supplementary Figure 4D.
Relative abundance of VSIG4 isoforms in cancer tissues (TCGA)
‘Long’ (Q9Y279-1)
‘Short’ (Q9Y279-3)
‘Long’ w/o C-tail (Q9Y279-2)
median
8

## Slide 9
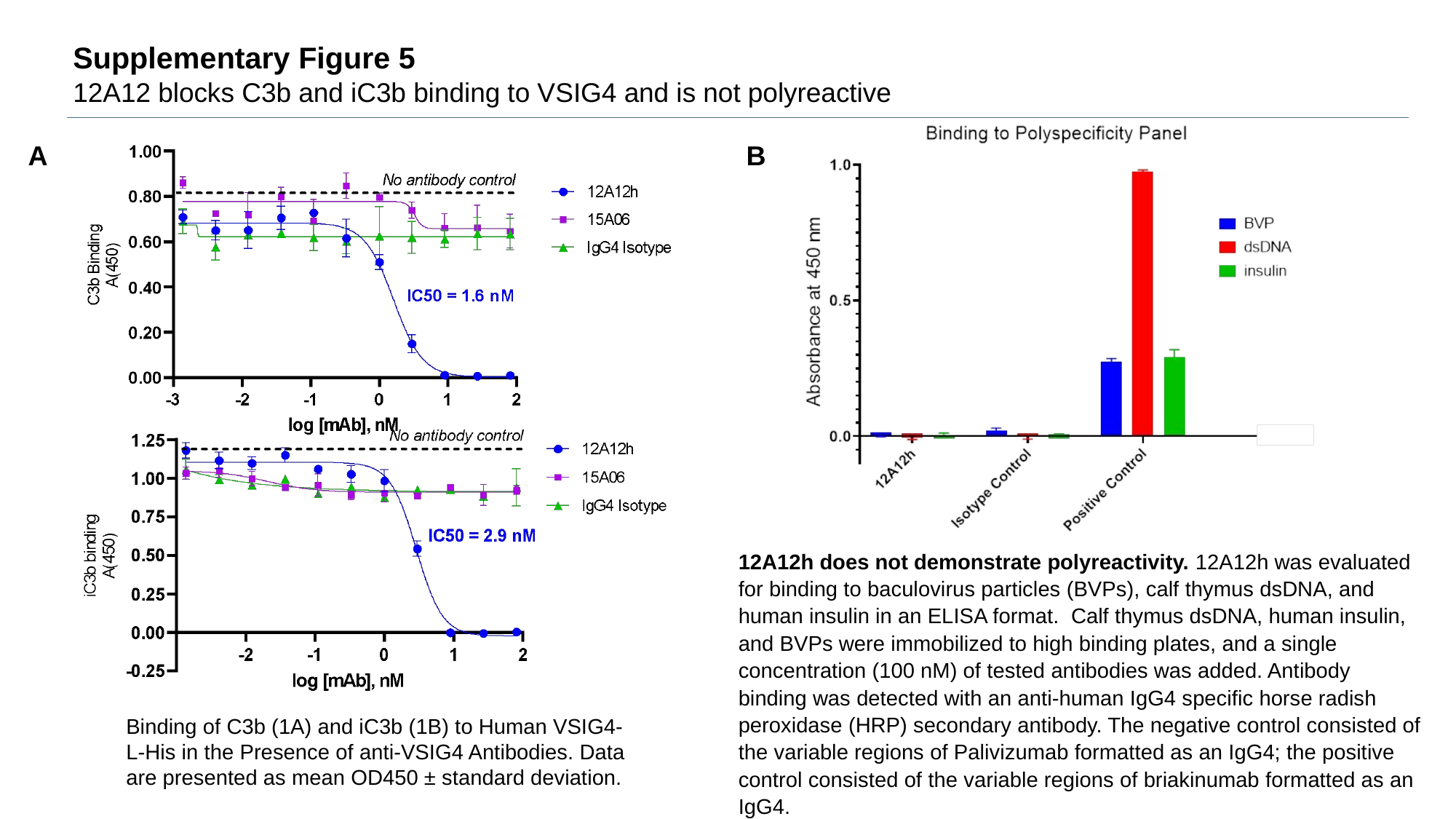

Supplementary Figure 5
12A12 blocks C3b and iC3b binding to VSIG4 and is not polyreactive
A
B
12A12h does not demonstrate polyreactivity. 12A12h was evaluated for binding to baculovirus particles (BVPs), calf thymus dsDNA, and human insulin in an ELISA format. Calf thymus dsDNA, human insulin, and BVPs were immobilized to high binding plates, and a single concentration (100 nM) of tested antibodies was added. Antibody binding was detected with an anti-human IgG4 specific horse radish peroxidase (HRP) secondary antibody. The negative control consisted of the variable regions of Palivizumab formatted as an IgG4; the positive control consisted of the variable regions of briakinumab formatted as an IgG4.
Binding of C3b (1A) and iC3b (1B) to Human VSIG4-L-His in the Presence of anti-VSIG4 Antibodies. Data are presented as mean OD450 ± standard deviation.

## Slide 10
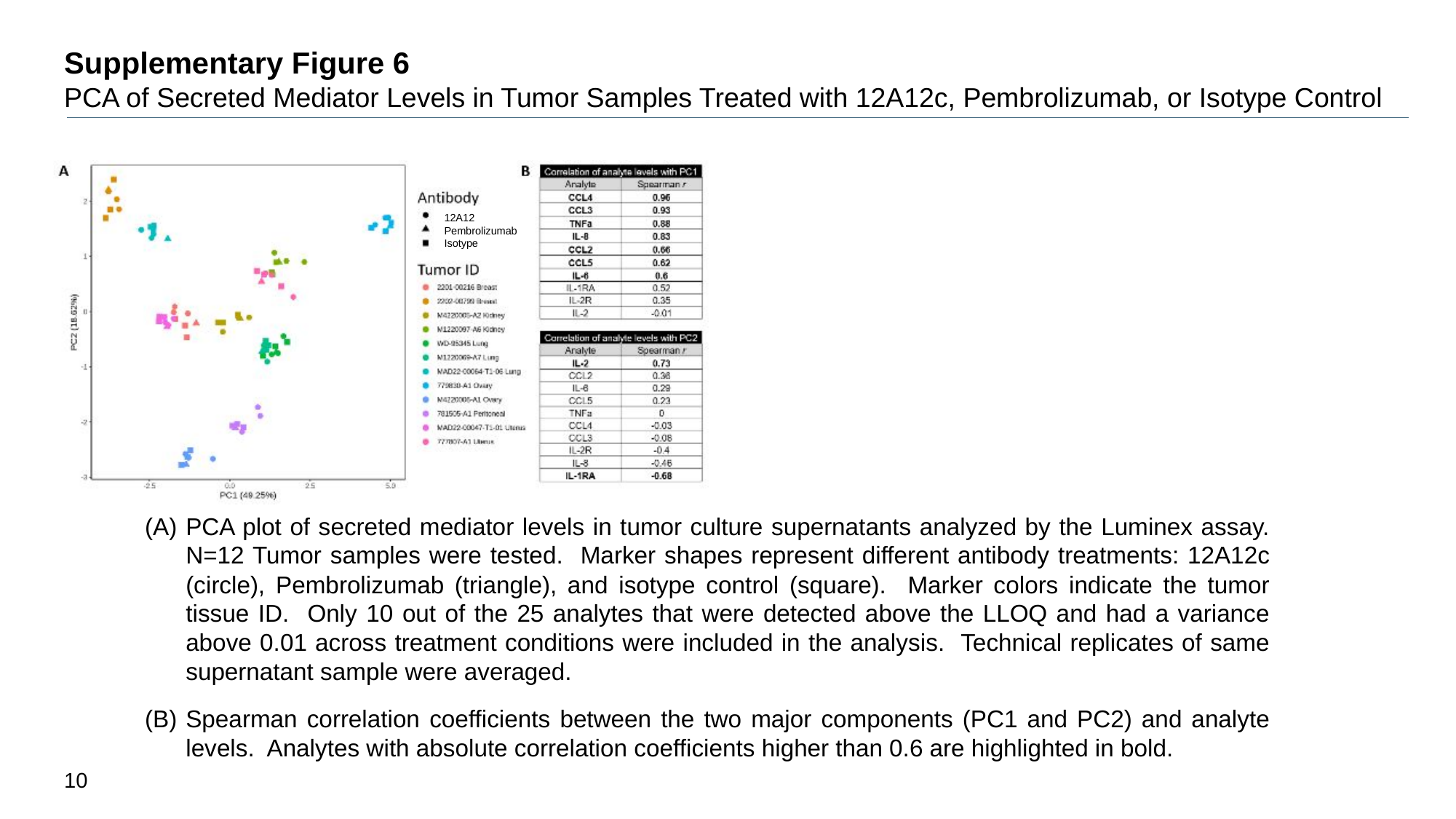

# Supplementary Figure 6 PCA of Secreted Mediator Levels in Tumor Samples Treated with 12A12c, Pembrolizumab, or Isotype Control
12A12
Pembrolizumab
Isotype
PCA plot of secreted mediator levels in tumor culture supernatants analyzed by the Luminex assay. N=12 Tumor samples were tested. Marker shapes represent different antibody treatments: 12A12c (circle), Pembrolizumab (triangle), and isotype control (square). Marker colors indicate the tumor tissue ID. Only 10 out of the 25 analytes that were detected above the LLOQ and had a variance above 0.01 across treatment conditions were included in the analysis. Technical replicates of same supernatant sample were averaged.
Spearman correlation coefficients between the two major components (PC1 and PC2) and analyte levels. Analytes with absolute correlation coefficients higher than 0.6 are highlighted in bold.
10

## Slide 11
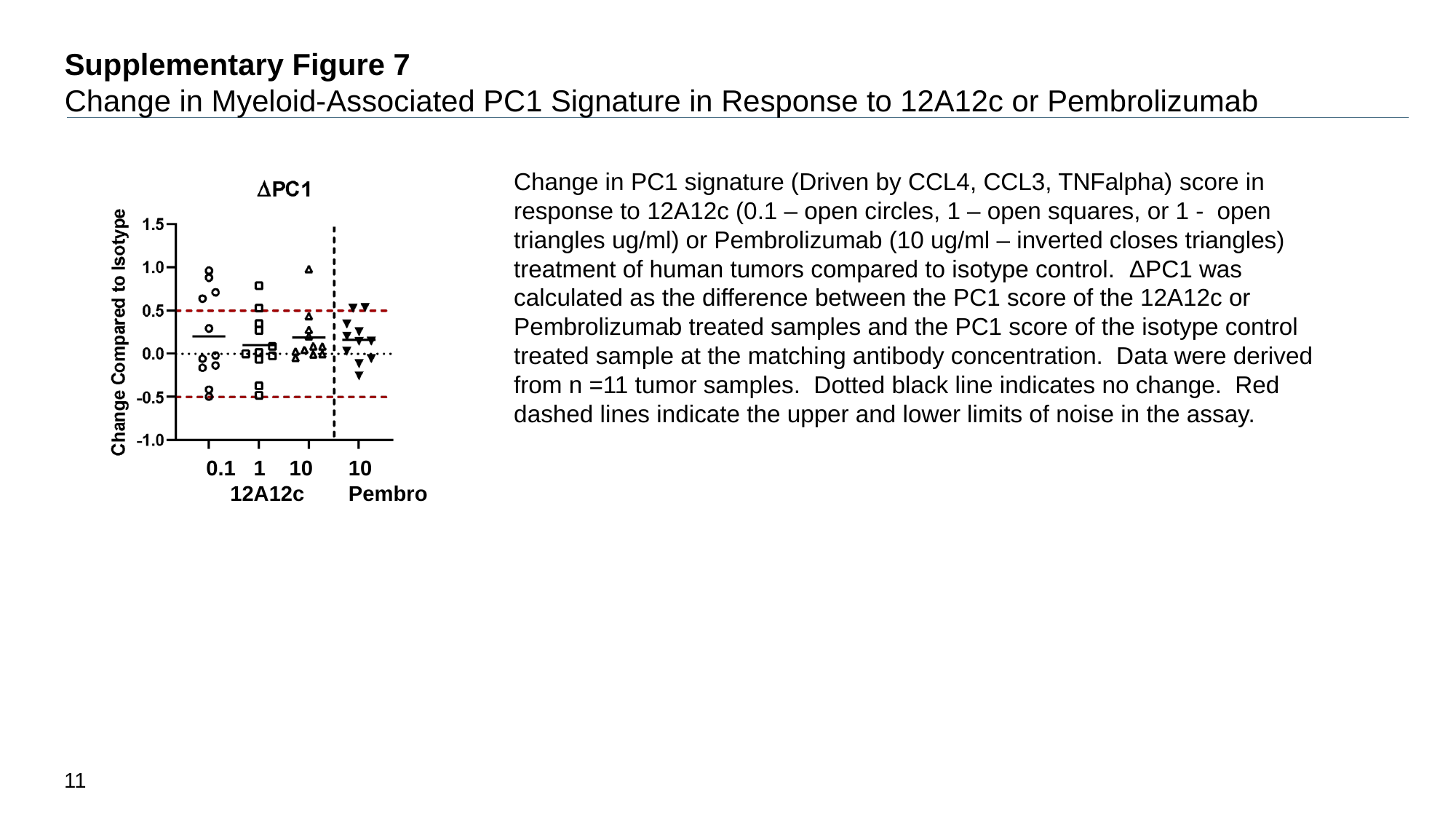

# Supplementary Figure 7 Change in Myeloid-Associated PC1 Signature in Response to 12A12c or Pembrolizumab
0.1 1 10
 12A12c
10
Pembro
Change in PC1 signature (Driven by CCL4, CCL3, TNFalpha) score in response to 12A12c (0.1 – open circles, 1 – open squares, or 1 - open triangles ug/ml) or Pembrolizumab (10 ug/ml – inverted closes triangles) treatment of human tumors compared to isotype control. ΔPC1 was calculated as the difference between the PC1 score of the 12A12c or Pembrolizumab treated samples and the PC1 score of the isotype control treated sample at the matching antibody concentration. Data were derived from n =11 tumor samples. Dotted black line indicates no change. Red dashed lines indicate the upper and lower limits of noise in the assay.
11

## Slide 12
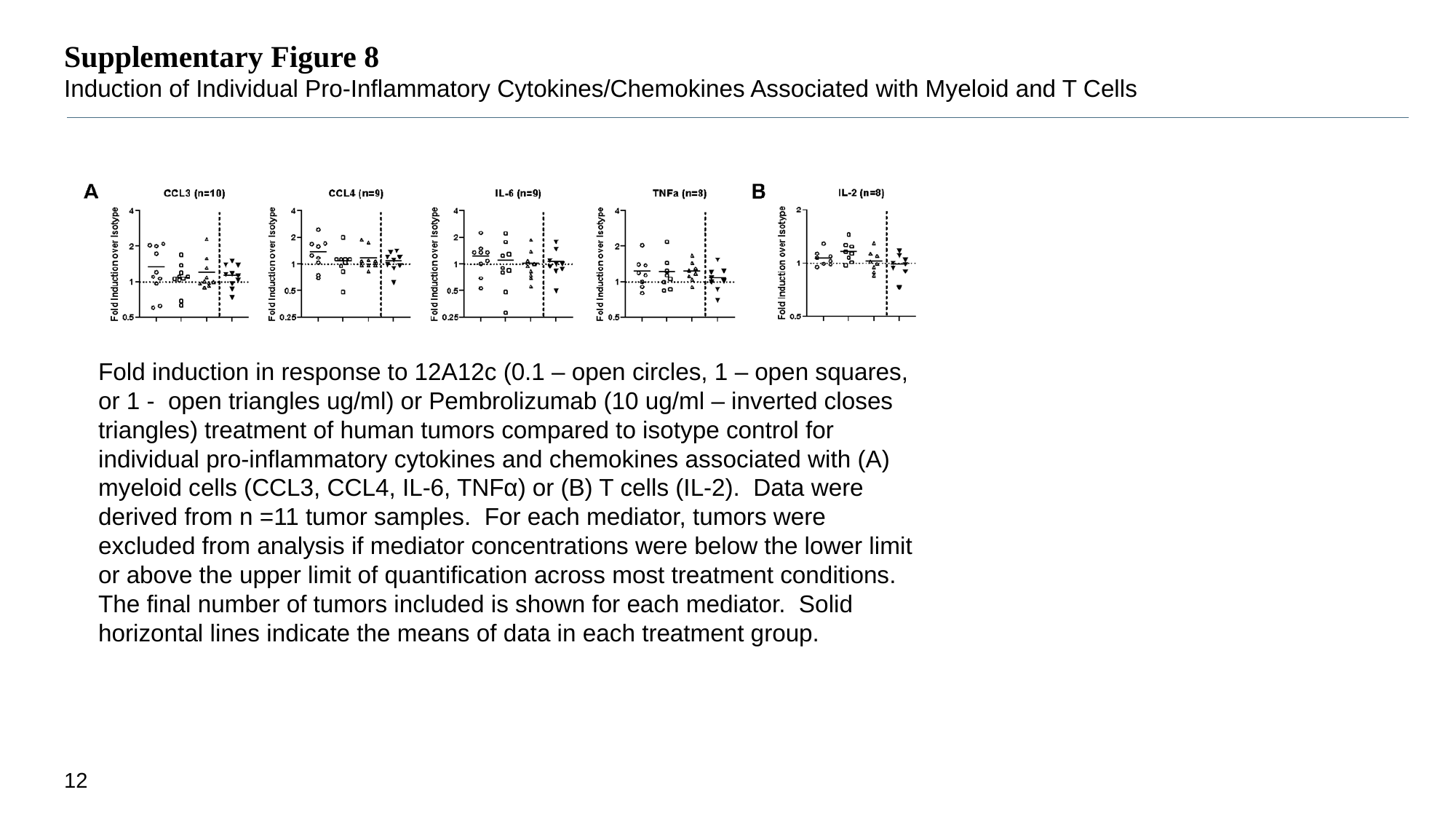

# Supplementary Figure 8 Induction of Individual Pro-Inflammatory Cytokines/Chemokines Associated with Myeloid and T Cells
Fold induction in response to 12A12c (0.1 – open circles, 1 – open squares, or 1 - open triangles ug/ml) or Pembrolizumab (10 ug/ml – inverted closes triangles) treatment of human tumors compared to isotype control for individual pro-inflammatory cytokines and chemokines associated with (A) myeloid cells (CCL3, CCL4, IL-6, TNFα) or (B) T cells (IL-2). Data were derived from n =11 tumor samples. For each mediator, tumors were excluded from analysis if mediator concentrations were below the lower limit or above the upper limit of quantification across most treatment conditions. The final number of tumors included is shown for each mediator. Solid horizontal lines indicate the means of data in each treatment group.
12

## Slide 13
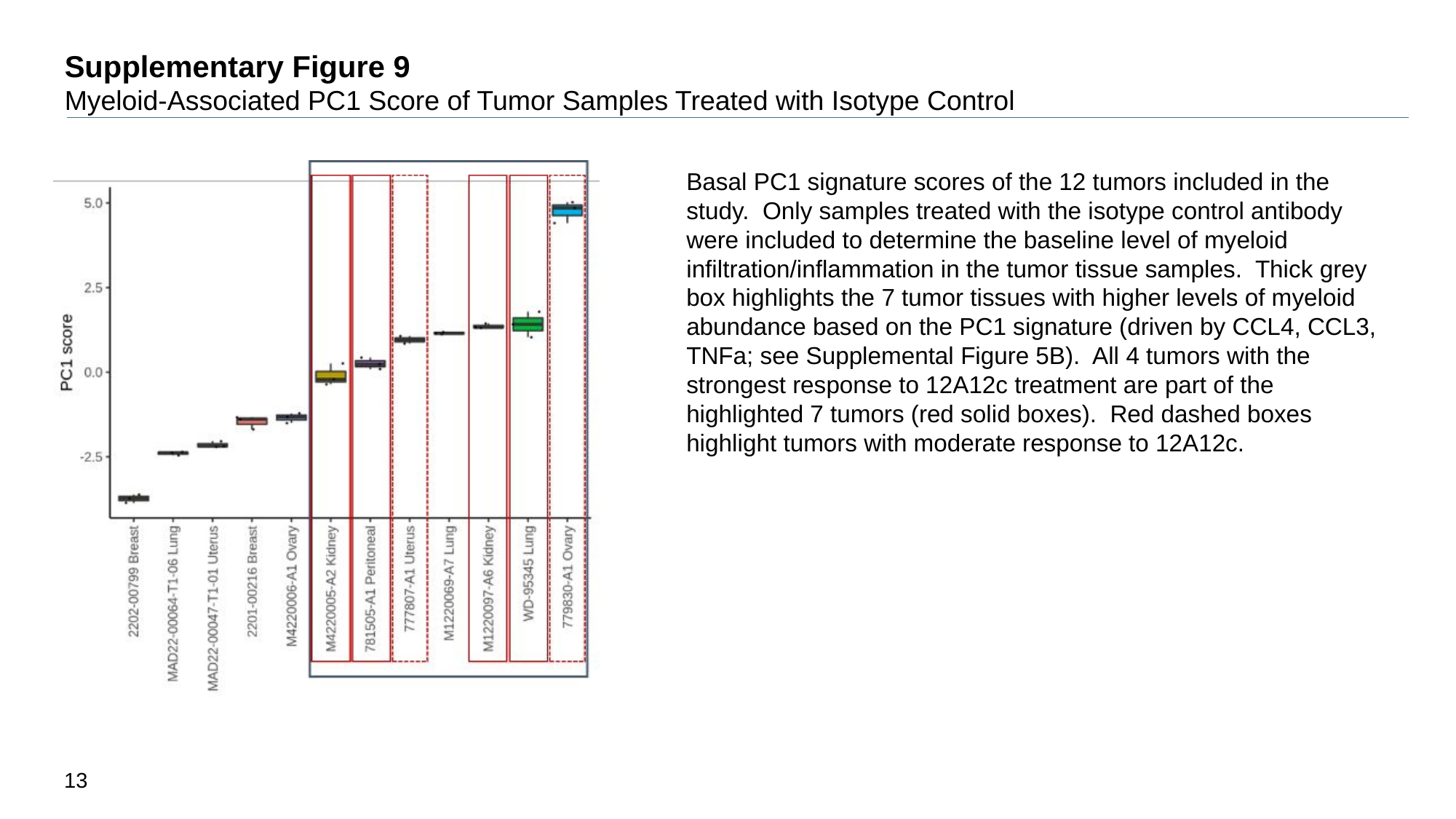

# Supplementary Figure 9 Myeloid-Associated PC1 Score of Tumor Samples Treated with Isotype Control
Basal PC1 signature scores of the 12 tumors included in the study. Only samples treated with the isotype control antibody were included to determine the baseline level of myeloid infiltration/inflammation in the tumor tissue samples. Thick grey box highlights the 7 tumor tissues with higher levels of myeloid abundance based on the PC1 signature (driven by CCL4, CCL3, TNFa; see Supplemental Figure 5B). All 4 tumors with the strongest response to 12A12c treatment are part of the highlighted 7 tumors (red solid boxes). Red dashed boxes highlight tumors with moderate response to 12A12c.
13

## Slide 14
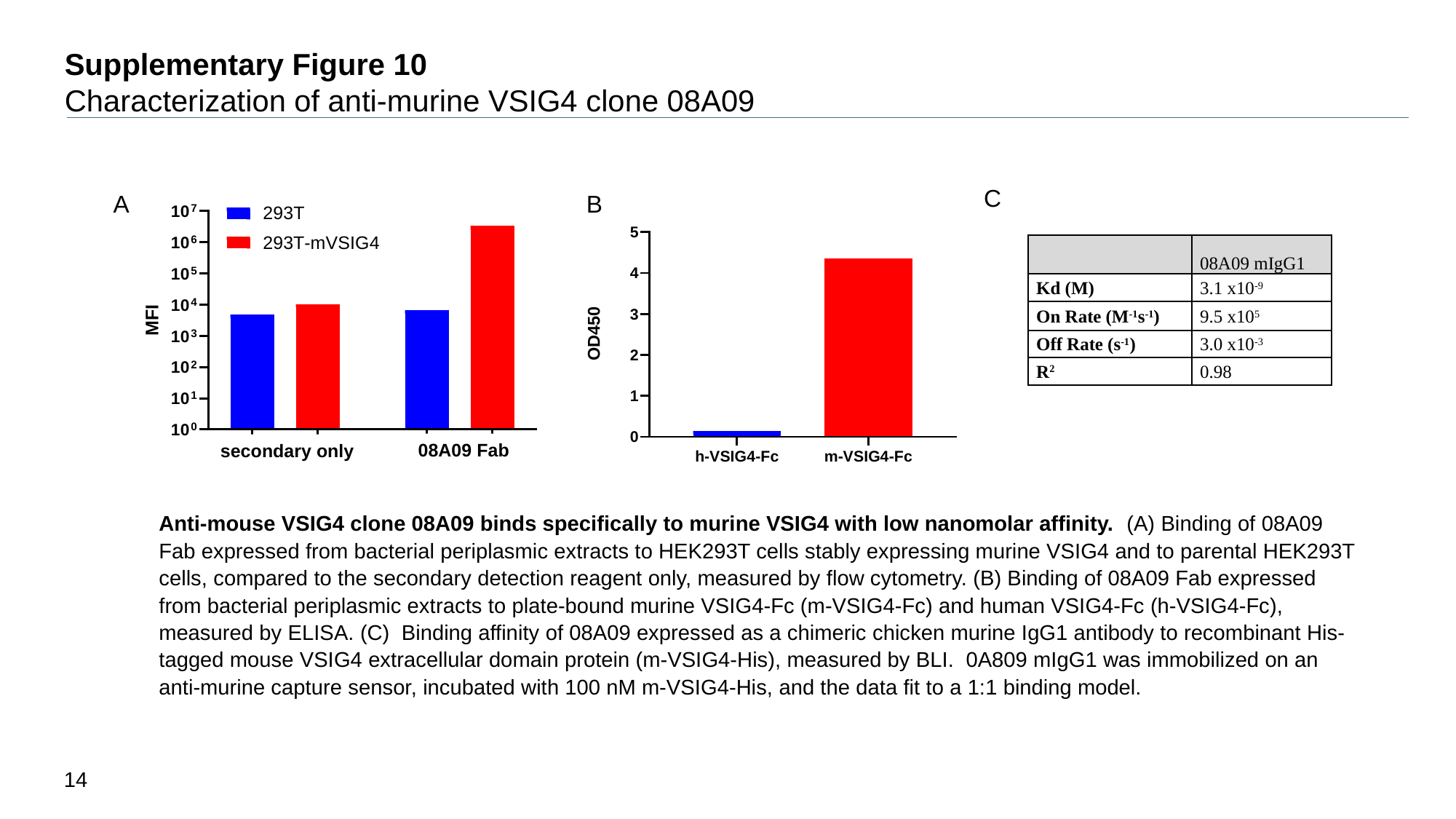

# Supplementary Figure 10 Characterization of anti-murine VSIG4 clone 08A09
C
A
B
| | 08A09 mIgG1 |
| --- | --- |
| Kd (M) | 3.1 x10^-9^ |
| On Rate (M-1s-1) | 9.5 x10^5^ |
| Off Rate (s-1) | 3.0 x10^-3^ |
| R2 | 0.98 |
08A09 Fab
secondary only
Anti-mouse VSIG4 clone 08A09 binds specifically to murine VSIG4 with low nanomolar affinity. (A) Binding of 08A09 Fab expressed from bacterial periplasmic extracts to HEK293T cells stably expressing murine VSIG4 and to parental HEK293T cells, compared to the secondary detection reagent only, measured by flow cytometry. (B) Binding of 08A09 Fab expressed from bacterial periplasmic extracts to plate-bound murine VSIG4-Fc (m-VSIG4-Fc) and human VSIG4-Fc (h-VSIG4-Fc), measured by ELISA. (C) Binding affinity of 08A09 expressed as a chimeric chicken murine IgG1 antibody to recombinant His-tagged mouse VSIG4 extracellular domain protein (m-VSIG4-His), measured by BLI. 0A809 mIgG1 was immobilized on an anti-murine capture sensor, incubated with 100 nM m-VSIG4-His, and the data fit to a 1:1 binding model.
14

## Slide 15
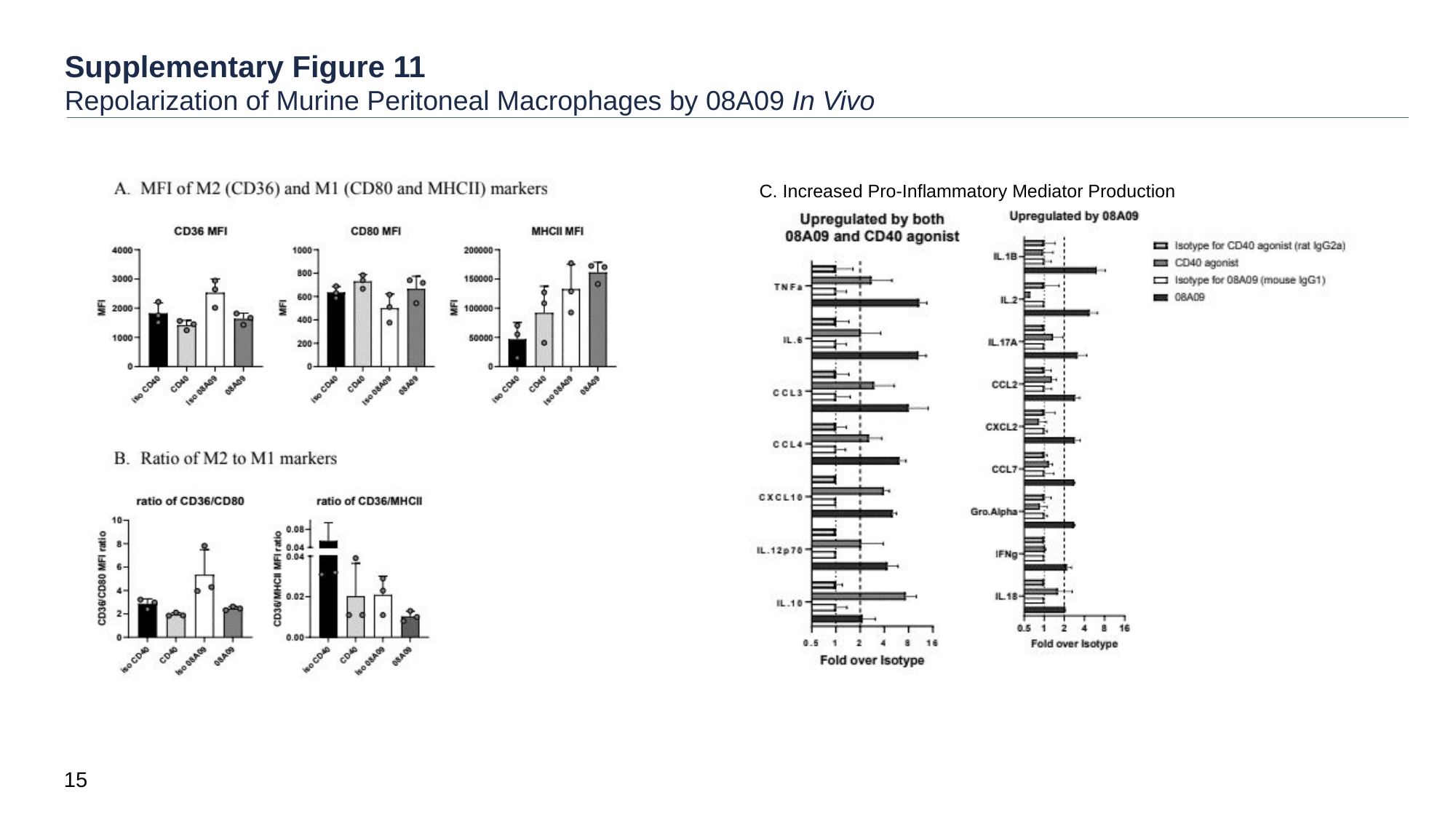

# Supplementary Figure 11 Repolarization of Murine Peritoneal Macrophages by 08A09 In Vivo
C. Increased Pro-Inflammatory Mediator Production
15

## Slide 16
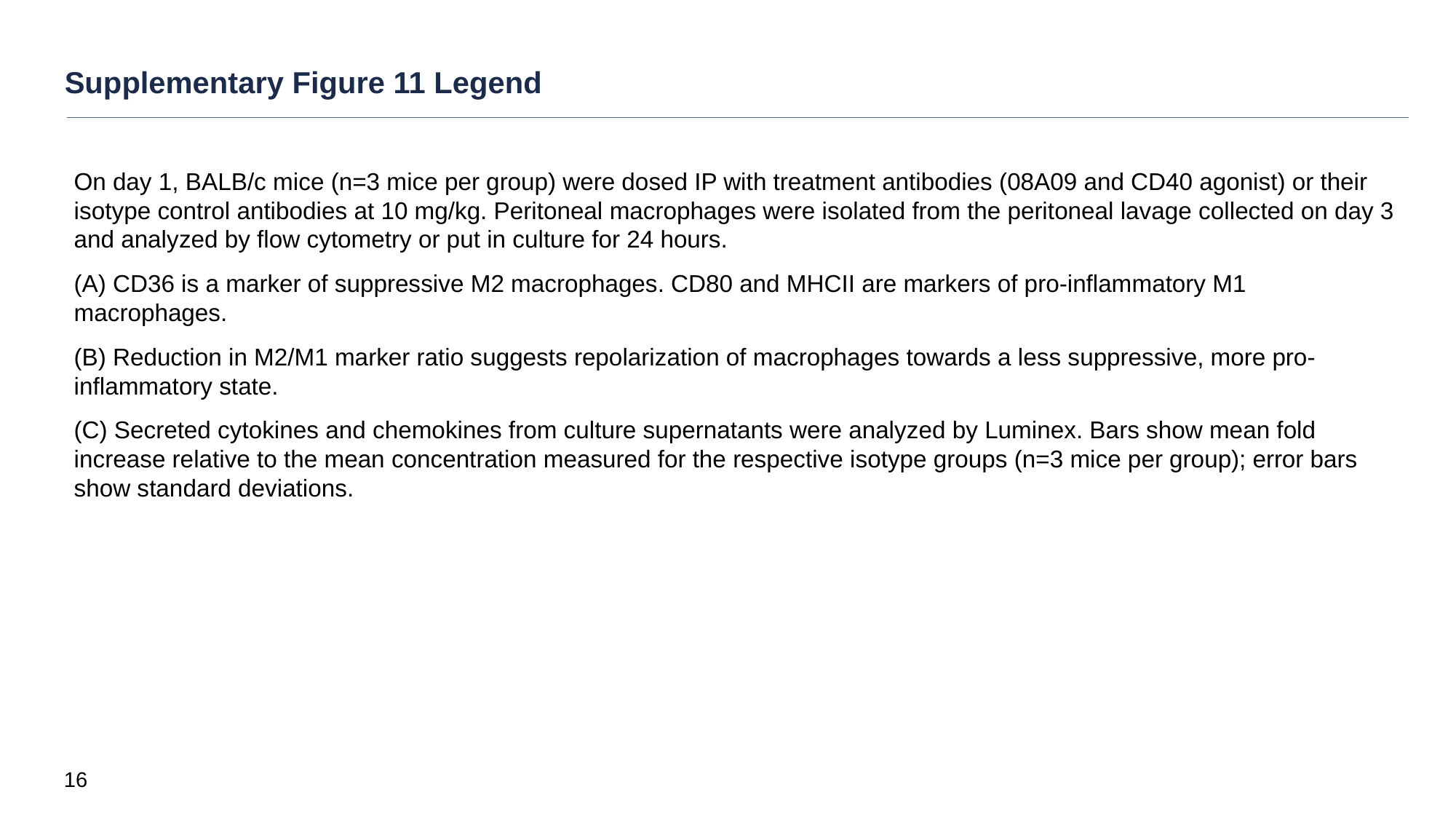

# Supplementary Figure 11 Legend
On day 1, BALB/c mice (n=3 mice per group) were dosed IP with treatment antibodies (08A09 and CD40 agonist) or their isotype control antibodies at 10 mg/kg. Peritoneal macrophages were isolated from the peritoneal lavage collected on day 3 and analyzed by flow cytometry or put in culture for 24 hours.
(A) CD36 is a marker of suppressive M2 macrophages. CD80 and MHCII are markers of pro-inflammatory M1 macrophages.
(B) Reduction in M2/M1 marker ratio suggests repolarization of macrophages towards a less suppressive, more pro-inflammatory state.
(C) Secreted cytokines and chemokines from culture supernatants were analyzed by Luminex. Bars show mean fold increase relative to the mean concentration measured for the respective isotype groups (n=3 mice per group); error bars show standard deviations.
16

## Slide 17
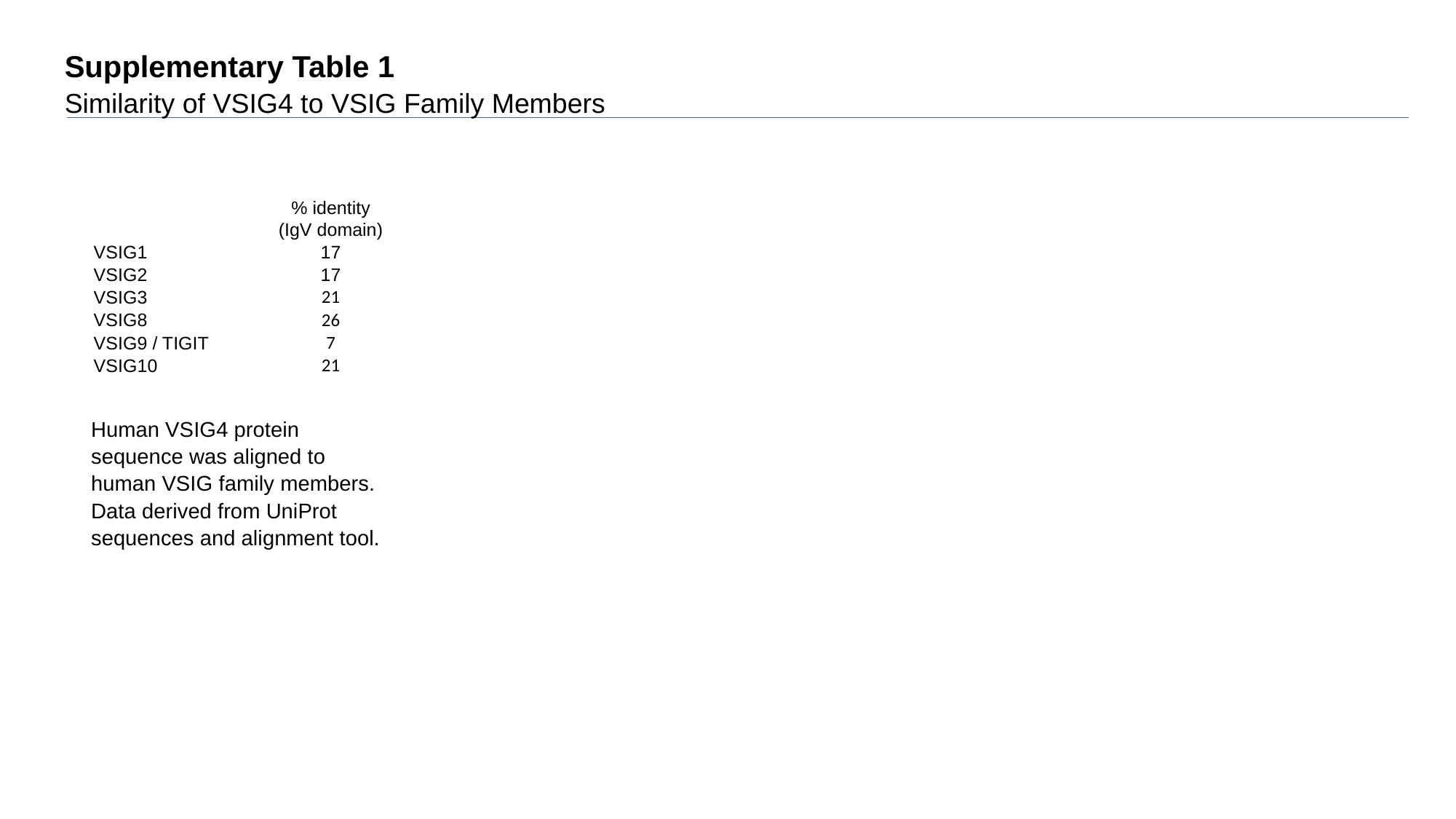

# Supplementary Table 1 Similarity of VSIG4 to VSIG Family Members
| | % identity (IgV domain) |
| --- | --- |
| VSIG1 | 17 |
| VSIG2 | 17 |
| VSIG3 | 21 |
| VSIG8 | 26 |
| VSIG9 / TIGIT | 7 |
| VSIG10 | 21 |
Human VSIG4 protein sequence was aligned to human VSIG family members. Data derived from UniProt sequences and alignment tool.

## Slide 18
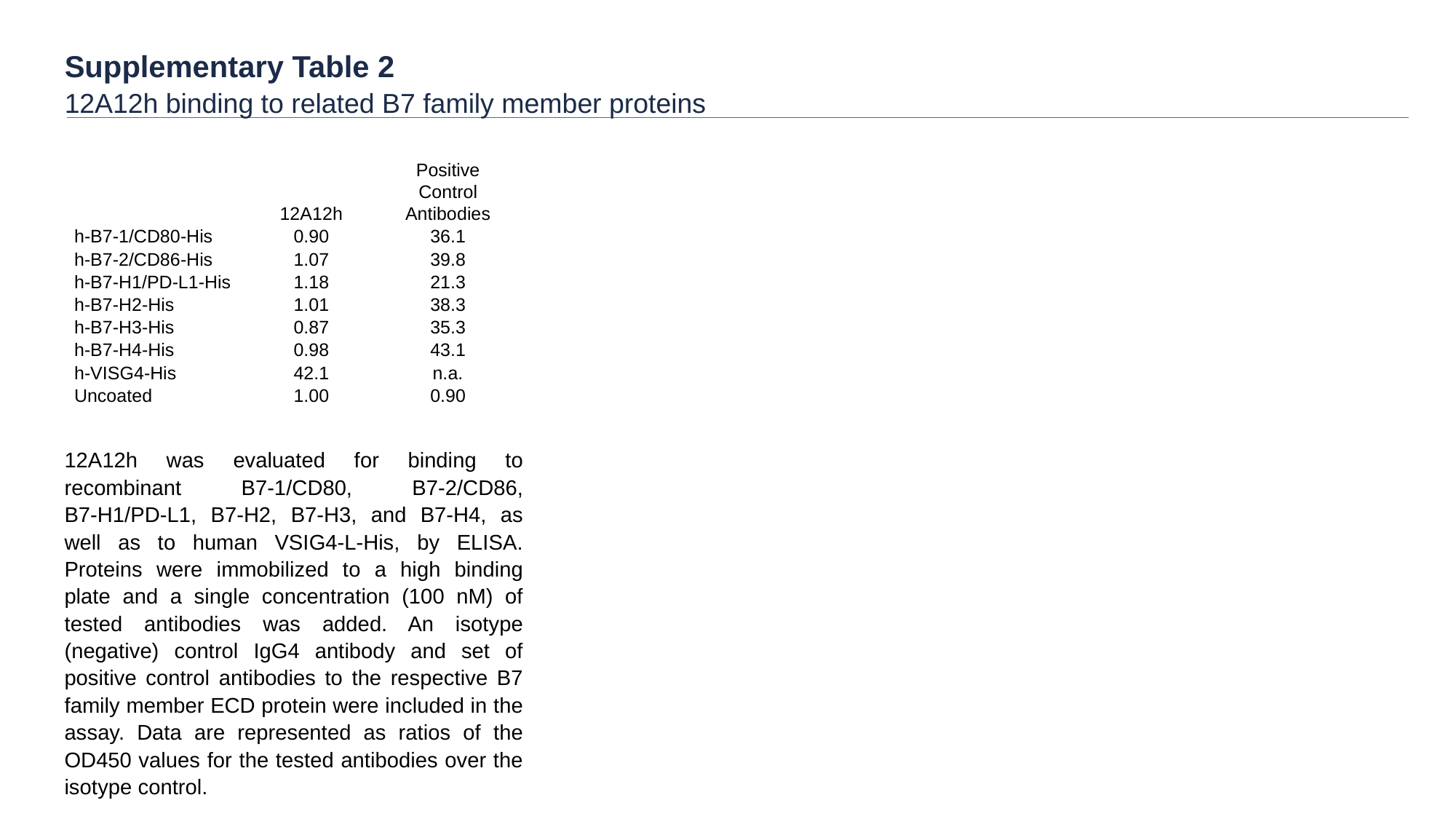

# Supplementary Table 2 12A12h binding to related B7 family member proteins
| | 12A12h | Positive Control Antibodies |
| --- | --- | --- |
| h-B7-1/CD80-His | 0.90 | 36.1 |
| h-B7-2/CD86-His | 1.07 | 39.8 |
| h-B7-H1/PD-L1-His | 1.18 | 21.3 |
| h-B7-H2-His | 1.01 | 38.3 |
| h-B7-H3-His | 0.87 | 35.3 |
| h-B7-H4-His | 0.98 | 43.1 |
| h-VISG4-His | 42.1 | n.a. |
| Uncoated | 1.00 | 0.90 |
12A12h was evaluated for binding to recombinant B7-1/CD80, B7-2/CD86, B7-H1/PD-L1, B7-H2, B7-H3, and B7-H4, as well as to human VSIG4-L-His, by ELISA. Proteins were immobilized to a high binding plate and a single concentration (100 nM) of tested antibodies was added. An isotype (negative) control IgG4 antibody and set of positive control antibodies to the respective B7 family member ECD protein were included in the assay. Data are represented as ratios of the OD450 values for the tested antibodies over the isotype control.
